# Emergence of *Echinococcus multilocularis* in Central Continental Croatia: A Human Case Series and Update on Prevalence in Foxes

**DOI:** 10.3390/life13061402

**Published:** 2023-06-16

**Authors:** Mirjana Balen Topić, Neven Papić, Klaudija Višković, Mario Sviben, Tajana Filipec Kanižaj, Stipislav Jadrijević, Daria Jurković, Relja Beck

**Affiliations:** 1 University Hospital for Infectious Diseases “Dr. Fran Mihaljević”, 10000 Zagreb, Croatia; npapic@bfm.hr (N.P.); kviskovic@bfm.hr (K.V.); 2 School of Medicine, University of Zagreb, 10000 Zagreb, Croatia; mario.sviben@hzjz.hr (M.S.); tajana.filipec.kanizaj@kb-merkur.hr (T.F.K.); stipislav.jadrijevic@kb-merkur.hr (S.J.); 3 Faculty of Health Studies, University of Rijeka, 51000 Rijeka, Croatia; 4 Department for Parasitology, Croatian Institute of Public Health, 10000 Zagreb, Croatia; 5 Merkur University Hospital, 10000 Zagreb, Croatia; 6 Department for Bacteriology and Parasitology, Croatian Veterinary Institute, 10000 Zagreb, Croatia; jurkovic@veinst.hr (D.J.); beck@veinst.hr (R.B.)

**Keywords:** human alveolar echinococcosis, *Echinococcus multilocularis*, epidemiology, clinical characteristic, red fox, emergence, Croatia

## Abstract

Human alveolar echinococcosis (HAE), caused by the metacestode stage of *Echinococcus multilocularis*, has emerged in many European countries over the last two decades. Here, we report the first data on the new HAE focus with increasing incidence in central Croatia, describe its clinical presentation and outcomes in diagnosed patients, and provide an update on the prevalence and geographic distribution of *Echinococcus multilocuaris* in red foxes. After the initial case in 2017 from the eastern state border, from 2019 to 2022, five new autochthonous HAE cases were diagnosed, all concentrated in the Bjelovar-Bilogora County (the county incidence in 2019 and 2021: 0.98/10^5^, in 2022: 2.94/10^5^/year; prevalence for 2019–2022: 4.91/10^5^). The age range among four female and two male patients was 37–67 years. The patients’ liver lesions varied in size from 3.1 to 15.5 cm (classification range: P2N0M0–P4N1M0), and one patient had dissemination to the lungs. While there were no fatalities, postoperative complications in one patient resulted in liver transplantation. In 2018, the overall prevalence of red foxes was 11.24% (28/249). A new focus on HAE has emerged in central continental Croatia, with the highest regional incidence in Europe. Screening projects among residents and the implementation of veterinary preventive measures following the One Health approach are warranted.

## 1. Introduction

The causative agent of human alveolar echinococcosis (HAE)—*Echinococcus multilocularis*—was described for the first time as a different species (Cestoda: Taeniidae) by Leuckart in 1863. Although rare, HAE is one of the most pathogenic zoonoses in Europe. If untreated, it is associated with high mortality, up to 90%, within 10–15 years of diagnosis [1]. While humans are an accidental intermediate host (metacestode stage of infection), red foxes (*Vulpes vulpes*), as definitive hosts, present the main source of infection.

Recently, a geographical spread of HAE across Europe and an increase in case numbers has been reported. Moreover, from 2001 to 2018, nine European countries reported the first case of autochthonous HAE [2]. Although endemic in neighboring Slovenia and Hungary, Croatia was considered HAE-free until the first case was diagnosed from Vukovar, on the eastern Croatian border, in 2017 and published in 2020 [3]. *E. multilocularis* infection in red foxes in Croatia has been reported previously, with a mean prevalence of 7.5% and 6.6% in 2015 and 2016, respectively [4].

Croatia is administratively divided into 21 counties, among which, with 2.652 square km located in central continental Croatia, the Bjelovar-Bilogora County occupies 3.03% of the whole state territory. Here, we report data on the new focus and increasing incidence of HAE in Bjelovar-Bilogora County; we analyzed disease outcomes and can provide an update on the prevalence and geographic distribution of the parasite among red foxes. A significant increase in its mean prevalence in red foxes has been noticed, especially in the County where new human cases appeared recently, suggesting the spread of this disease from eastern to central continental Croatia, where one of the southernmost focuses of HAE in the Balkan region is emerging. Educational and screening projects among residents and the implementation of veterinary preventive measures should be initiated to prevent the spreading and decrease morbidity of this serious parasitic zoonosis.

## 2. Materials and Methods

### 2.1. Human Alveolar Echinococcosis

Patients included in this study were treated at the University Hospital for Infectious Diseases in Zagreb, which is a national referral center for infectious diseases. One additional patient from Split (Dalmatia) was diagnosed with HAE in the period studied but was not included in this case series since the disease was not autochthonous but probably imported from South Germany. According to the official data from the Croatian Institute of Public Health, there were no other patients with a diagnosis of AE reported in Croatia up to April 2023. The patientsʹ data were collected from medical documentation and analyzed retrospectively. The disease was classified based on morphological characteristics of the lesions obtained by multi-slice computed tomography (MSCT) using the echinococcosis PNM staging system [5].

The disease incidence and prevalence data for Bjelovar-Bilogora County were recalculated based on a population of 101,879 inhabitants, according to a recent population census [6].

In all patients, the diagnosis was confirmed by the PCR and sequencing of two gene targets either from formalin-fixed, paraffin-embedded liver tissue or from fresh cysts collected during explorative surgery. Paraffin-embedded tissue was dewaxed with xylene, washed three times with 99.6% ethanol, and digested at 56 °C for 8 h, while, from fresh cysts, this step was skipped. DNA was extracted using a QIAamp DNA Mini QIAcube Kit according to the protocol for blood and body fluids on an automated QIAcube system.

In patient No 1, serology (genus and species-specific ELISAs and EUROLINE-IgG Western Blot system) was performed at the University of Zürich (Zürich, Switzerland) [3], while other patients were serologically tested at the Croatian Institute of Public Health, Department of Parasitology (Zagreb, Croatia). In patient No 2, NovaLisa *Echinococcus* IgG ELISA was used for the qualitative determination of IgG class antibodies against *Echinococcus* spp. in human serum or plasma (manufacturer NovaTec Immundiagnostica GmbH, Dietzenbach, Germany; by manufacturer-declared sensitivity: 97.22%, and specificity: 98.82%). In addition, an *Echinococcus* Western Blot IgG (manufacturer LDBIO Diagnostics, Lyon, France), a qualitative test for the serological diagnosis of alveolar and hydatid echinococcosis, was used as a confirmatory test. In patients No 3–6, besides the aforementioned tests, an *E. multilocularis* ELISA test (manufacturer Bordier Affinity Products, Crissier, Switzerland; by manufacturer declared sensitivity: 83%, and specificity: 98%) was used for the quantitative detection of IgG antibodies against Em2 and Em18 specific antigens of *E. multilocularis.*

### 2.2. E. multilocularis Infection in Red Foxes

Fecal samples were collected from 249 carcasses of red foxes, as part of the rabies control program in different regions of Croatia, from January to March 2018. Carcasses were delivered to the veterinary services, and the approximate location, based on the hunters’ mandatory reporting, was recorded for each sample. After collection, fecal samples were stored at −80 °C for at least 3 weeks prior to further processing. DNA was extracted directly from 200 mg of fecal samples using the QIAamp^®^ DNA Stool Mini Kit (Qiagen, Hildesheim, Germany). All samples were analyzed using conventional *E. multilocularis* PCR reactions that amplified a 200-bp region in the mitochondrial gene nad1 [7] or a 395-bp region in the 12S rRNA gene [8]. Amplicons were purified, sequenced in both directions with the same primers as used in PCRs, assembled using SeqMan Pro, and edited with EditSeq (Lasergene, DNASTAR).

**Figure 1:**
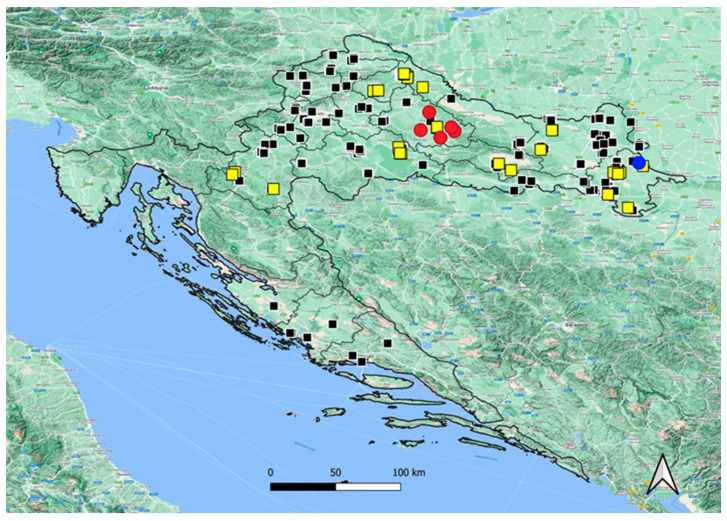
Geographic distribution of autochthonous human alveolar echinococcosis cases from 2017 to 2022, and distribution of localities with *Echinococcus multilocularis* positive red foxes (*Vulpes vulpes*), tested from January to March 2018 in Croatia. Yellow squares—locations of positive foxes; Black squares—locations of negative foxes; Blue dot—first human case from 2017; Red dots—positive human cases from 2019 to 2022.

For better visibility of the obtained results, all samples were mapped using QGIS software version 3.30.0 RC, and Figure 1 was created using the QGIS program [9].

## 3. Results

### 3.1. Human Alveolar Echinococcosis

In 2017, the first case of HAE was diagnosed in Croatia. From 2019 to 2022, five new autochthonous HAE cases were diagnosed, all concentrated in the rural eastern part of Bjelovar-Bilogora County in central continental Croatia (Figure 1). The incidence in this County in 2019 and 2021 was 0.98/10^5^/year, and it rose to 2.94/10^5^/year in 2022. In 2020 there were no cases recorded. The County prevalence for 2019–2022 was 4.91/10^5^.

The basic demographic and clinical characteristics of the diagnosed autochthonous HAE patients are shown in Table 1.

All HAE patients were from rural areas and were exposed to rural soil and home garden-grown vegetables near the wood; however, some of them were additionally engaged in forest activities (wood and mushroom collecting). The duration of symptoms before the final diagnosis ranged from two months to eight years. The patient’s liver lesions varied in size from 3.1 to 15.5 cm in diameter. The patient with the largest lesion (No 5) was followed up for 8 years under the diagnosis of “atypical hemangioma”. Only in patient No 1 was a spread in the infection spread found outside the liver to the lungs. All patients were surgically treated: two by partial resection (patient No 1 with the disseminated disease and No 4 with numerous parasitic lesions which permeated virtually the whole liver) and four by total excision. In three of the six patients, who were initially misdiagnosed as having a neoplastic tumor of the liver, preoperative albendazole therapy was not applied. One severe and two moderate reactions to albendazole were recorded, which led to albendazole discontinuation in three patients. The most serious adverse effect of albendazole occurred in patient No 1, with disseminated disease to the lungs, for whom one year after palliative surgery, albendazole had to be discontinued due to significant pancytopenia and sepsis triggered by agranulocytosis. In this patient, “salvage therapy” with amphotericin B (deoxycholate) was given three times weekly for four weeks and once a week for the next two years. Afterward, due to a rise in serum creatinine and gamma-glutamyl transferase, the therapy was switched to mefloquine, 1 × 250 mg once per week. After 1.5 years, mefloquine therapy had to be discontinued due to malaise, leucopenia, and a rise in serum creatinine and gamma-glutamyl transferase. Despite intolerance to multiple therapies, there were no signs of disease progression during the 5-year long follow-up period in this patient, and the last FDG-PET scan in March 2022 showed no metabolic activity in the remaining parasitic lesions. In patient No 3, the post-excisional clinical course was complicated by hepatic artery thrombosis, and consequent episodes of severe recurrent septic cholangitis were indications for liver transplantation, which was successfully performed. In our cohort, there were no fatal outcomes.

### 3.2. Prevalence of E. multilocularis Infection in Red Foxes

In the observed period from January to March 2018, the fecal samples of 249 red foxes from different parts of continental Croatia were examined by PCR for the presence of *E. multilocularis* genes. Among them, 28/249 samples were positive, with a calculated mean prevalence of 11.24% (95% CI: 7.89–15.77). An almost identical prevalence of 11.7% (6/51; 95% CI: 5.51–23.38) was found on location in Eastern Croatia, from where the first HAE case was diagnosed. In Bjelovar-Bilogora County, where new human cases recently emerged, local prevalence among red foxes reached 28.57% (2/7; 95% CI: 8.22–64.11). The locations from which the fecal samples of red foxes were analyzed and the distribution of locations with *E. multilocularis* positive and negative findings are shown in Figure 1.

Sequences from humans and red foxes obtained in the current study were identical to each and to Croatian sequences submitted earlier to GenBank under the accession numbers MG755265 and MG755266.

## 4. Discussion

The incidence for HAE of 2.94/10^5^/year in 2022 and prevalence of 4.91/10^5^ for 2019–2022 in Bjelovar-Bilogora County in central continental Croatia exceeded the overall incidence reported for Central Europe of 0.03–0.26/10^5^ [10], and the national mean incidence of 0.09/10^5^/year in our neighboring country Slovenia (2001–2005) [11]. It also exceeded the highest reported European regional incidence of 1.9/10^5^ in the Austrian Federal State of Vorarlberg in 2011 by more than double [12]. In addition to an increase in the red fox population in Europe, related to the elimination of rabies and their increased infection rates with *E. multilocularis*, the increasing urbanization of fox habitats has been proposed as one of the important factors associated with an increase in the incidence of HAE cases [2].

The high County prevalence of HAE in Bjelovar-Bilogora County is in accordance with a high observed County prevalence among red foxes of 28.57% recorded in 2018. As in Croatia, in the neighboring countries, a rise in *E. multilocularis* infection prevalence among red foxes has been observed: in Slovenia, it increased from 2.6% (2010) [13] to 29.1% (2019–2022) [14]. In Hungary, the mean prevalence of red foxes gradually increased, and it was 5%, 10.7%, 7.9%, and 12.5% in 2002, 2008–2009, 2012–2013, and 2018–2020, respectively [15,16,17]. In the Vojvodina Province of Serbia, a prevalence of 17.9% was recorded in 2016 [18].

Recently (2022), the first positive red fox was reported from Bosnia and Herzegovina (B&H) [19], caught close to the westernmost state border with Croatia, in a belt of the Dinaric Mountains that spread continuously from Slovenia to Albania and where from the Croatian territory two positive red foxes were reported in 2015–2016 [4].

In Croatian surroundings, autochthonous HAE cases were reported from southern and north-eastern parts of Slovenia—there were nine serologically and morphologically diagnosed cases between 2001 and 2005 [11]. In Hungary, a case series of 16 HAE patients (2003–2018) was published: four patients (25%) were cured by radical surgery and adjuvant albendazole therapy, five (31.3%) were unresectable but without progression under albendazole therapy, in seven (43.8%) the disease progressed, and there were three (18.8%) lethal outcomes recorded [20]. In comparison to these data, the data from our study suggest better disease outcomes (no disease progression, no lethal outcome) among our patients, although this conclusion could be biased due to a low number of cases. However, no reports of HAE from Serbia or B&H can be found in the literature yet, which could be the result of a potential misdiagnosis bias [19].

A meta-analysis from 2016 showed the highest pooled prevalence of the parasite among red foxes in Baltic and central European countries of 58.0, 36.8, 34.9, 29.2 and 27.3% in Lithuania (2001–2006), Latvia (2002–2008), Liechtenstein (1990–1992), Germany (2000–2012) and Slovakia (2000–2013), respectively [21]. Recent animal data from neighboring Slovenia [14] and data from Bjelovar-Bilogora County from this study, where parasite prevalence among examined red foxes in the observed regions reached nearly 30%, clearly show the spread of this infection in the main host; this has resulted in the local emergence of HAE cases in central continental Croatia. The low disease incidence and unfamiliarity of healthcare workers with alveolar echinococcosis have contributed to initial misdiagnosis in a substantial proportion of our patients.

## 5. Conclusions

Continental Croatia should be considered endemic for HAE. A new focus on HAE is emerging in Bjelovar-Bilogora County, where a high parasite prevalence in red foxes has been recently documented. The education of the general population and clinicians, screening projects among local inhabitants, the implementation of veterinary medicine preventive measures, and systematic surveillance of the parasite prevalence in red fox populations should be initiated.

## Figures and Tables

**Table 1:** Demographic and clinical characteristics of autochthonous human alveolar echinococcosis patients in Croatia from 2017 to 2022.

Patient: Gender, Age, Year of Diagnosis	Clinical Presentation (Duration of Symptoms before Diagnosis)	PNM Classification (Clinical Stage)	Diagnosis and Follow Up	Therapy	Complications
No 1: male; 63 y.; 2017	Fever, dyspnea, cough, right-sided chest pain (3 y.)	P4N1M1 (IV)	MSCT Pathohistology Serology PCR FDG-PET	Albendazole; Partial resection; Amphotericin B; Mefloquine	Intraop. bleeding; Pancytopenia, agranulocytosis with sepsis—due to albendazole therapy
No 2: male; 64 y.; 2019	Upper right abdominal quadrant pain; jaundice (5 mo.)	P4N1M0 (IV)	MSCT Pathohistology PCR	Liver lobectomy; Albendazole 2 y. postoperatively	No
No 3: female; 37 y.; 2021	Upper right abdominal quadrant pain (8 mo.)	P4N0M0 (IIIb)	MSCT Pathohistology Serology PCR FDG-PET	Excision; Albendazole postoperatively; Liver transplantation	Postexcisional: hepatic artery thrombosis; recurrent cholangitis
No 4: female; 58 y.; 2022	Upper right abdominal quadrant pain, nausea (2 mo.)	P4N0M0 (IIIb)	MSCT Pathohistology Serology PCR FDG-PET	Albendazole Partial resection	No
No 5: female; 67 y.; 2022	Upper right abdominal quadrant discomfort (8 y.)	P3N0M0 (IIIa)	MSCT Pathohistology Serology PCR FDG-PET	Liver lobectomy; Albendazole postoperatively	Leucopenia; Increased transaminases due to albendazole therapy
No 6: female; 53 y.; 2022	Upper abdominal discomfort, nausea (4 y.)	P2N0M0 (II)	MSCT Serology FDG-PET Pathohistology PCR	Albendazole; Excision	Increased transaminases and neutropenia due to albendazole therapy

y.—years; mo.—months; MSCT—Multi-Slice Computed Tomography; PCR—Polymerase Chain Reaction; FDG-PET—Fluorodeoxyglucose-Positron Emission Tomography.

## Data Availability

The data presented in this study are available on request from the corresponding author. Detailed patient data are not publicly available due to privacy or ethical restrictions.
